# Analogies evolve by increasing transmission fidelity in the communication of complex information

**DOI:** 10.1017/ehs.2026.10063

**Published:** 2026-07-21

**Authors:** Thomas Holding, Paul E. Smaldino, Charlotte O. Brand

**Affiliations:** 1 BirthRites Lise Meitner Research Group, Max-Planck-Institute for Evolutionary Anthropology, Leipzig, Germany; 2 Department of Cognitive and Information Sciences, University of California-Merced, Merced, New Mexico, USA; 3 Santa Fe Institute, Santa Fe, California, USA; 4 Complexity Science Hub, Vienna, Austria; 5 Human Behaviour and Cultural Evolution Group, University of Exeter College of Life and Environmental Sciences, Penryn, UK; 6 Department of Psychology, University of Sheffield, Sheffield, UK

**Keywords:** analogy, high fidelity, social learning, cultural evolution, NK landscape

## Abstract

Analogies are a fundamental part of our cognition and communication. Humans are also a social species with shared cultural repertoires learnt throughout our lifetimes. As such, we expect analogies to enable the learning of complex, novel information by communication that takes advantage of shared cultural information. Here, we demonstrate the plausibility of this proposal and clarify its scope through computational modelling. We first model the individual-level process of learning via analogy with access to a shared repertoire of cultural information, using NK landscapes to represent high-dimensional solution spaces for complex problems. We then analyse a model of population dynamics to consider the conditions under which analogical communication will evolve and be maintained when costly. Our analyses suggest that even when analogy use is costly in terms of both search time and memory constraints, it can nevertheless be advantageous by allowing learners to obtain high-quality solutions more efficiently and more often than those who do not learn via analogy.

## Graphical abstract

**Figure:**
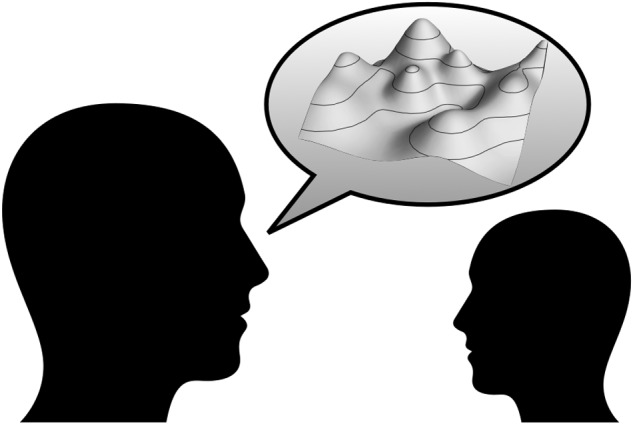

## Social media summary

Analogical communication using shared cultural repertoires results in high-fidelity social learning of complex problems.

## Introduction

The social learning of complex cultural behaviours is what makes the human species so successful and able to operate in a huge variety of ecological niches all over the planet (Boyd et al., 2011). Many of these cultural behaviours and technologies are extremely complex and require active teaching and attentive learning, often accompanied by verbal and gestural communication (Heyes, 2020; Laland et al., 2000; Sterelny, 2016). Models and experiments of social learning in humans often assume that humans can rely on high-fidelity copying, presumably via imitation of some kind. It is typically assumed that language is either totally absent and imitation occurs by observation, or that fully fledged language is available for transmission. Rarely do we see consideration of the crucial time in our evolutionary history in which language was *evolving*, during which there was a basic form of protolanguage that presumably aided communication but lacked the semantic and pragmatic flexibility of modern language (Fitch, 2010). This language gap is important, because high-fidelity transmission of complex information by language-free imitation is often highly error-prone (Thompson et al., 2022) and because complex tool use and teaching with language may have coevolved during this period of our evolutionary history (Fitch, 2010; Morgan et al., 2015).

We suggest that this crucial time would have witnessed the emergent use of analogies in human communication, where an analogy is defined as the explicit mapping of the relational structures of a novel stimulus to those of a known concept or familiar stimulus for the purpose of knowledge transfer (Brand et al., 2021; Gentner et al., 2001; Hofstadter & Sander, 2013; Holyoak & Thagard, 1995; Lupyan & Winter, 2018). The process of communicating via already-formed labels and categories necessarily improves learning and understanding of new and complex behaviours, and helps to explain why our modern language is littered with analogy, metaphor, and story. Analogical communication is not merely a by-product that came after the development of language, but is likely to have played a key role in facilitating how we cognitively process and communicate about our environment at the most basic level (Brand et al., 2021; Hofstadter & Sander, 2013). The evolution of analogical communication need not have required specialised genetic adaptations; our hypothesis is consistent with cultural evolutionary approaches that describe processes by which the nature of language was facilitated by pre-existing adaptations for social learning, social tolerance, and cooperation (Chater & Christiansen, 2010; Kirby, 2017; Thompson et al., 2016; Tomasello et al., 2012).

Analogical *thinking* allows individuals to draw upon similarities between different systems or situations to aid the search for insight and understanding, and to compress information into more easily encoded and recalled chunks (Bod, 2009; Hofstadter & Sander, 2013; Schustack & Anderson, 1979). Analogical *communication* works similarly, but involves a critical asymmetry in terms of information held by a teacher and a learner. The teacher has new information or know-how that they wish to communicate to the learner. Simple information can of course be communicated without analogy, or even words. Pointing to an apple on a tree branch directs the learner’s attention to the branch, while an expression of fear followed by frantic running away informs the learner that something dangerous is approaching! It is well understood how simple conventions mapping signals to prescribed actions can develop in cooperative communities (Lewis, 1969; Skyrms, 2010). Communicating complex knowledge and know-how, on the other hand, is much more difficult, even with a full lexical toolkit, if one must speak directly and literally (Miton & DeDeo, 2022). This is illustrated by the difficulty often faced by novice computer programmers in providing instructions to a machine that can neither interpolate nor draw on similarities to related situations (Cleland, 2001).

Experienced communicators (and mediocre golfers) can say things like ‘Grip the club lightly like a baby bird; not so tight that you strangle it but tight enough so it doesn’t fly away.’ These instructions rely on shared knowledge of the fragility and skittish nature of baby birds. Delivering similar instructions without this reference to shared cultural knowledge would require much more laboured speech and communication of tacit knowledge, which is, by definition, impossible to communicate. In our example, communicating grip strength would require expertise in a measurement of grip strength that can only be understood by experience and cannot be known by the teacher and would result in something no more efficient than trial and error by the student, e.g., ‘securely but not too tightly … a little looser, no a little tighter …’. Even this more ‘literal’ explicit communication requires the use of labels and concepts such as ‘securely’, ‘tighter/looser’. Some cognitive scientists, particularly Hofstadter and Sander (2013), have argued that *all* categorical labels should be considered analogies, because they only make sense in terms of their similarity to previously encountered examples with the same label. In this sense, and in contrast to narrower definitions requiring mapping of deep relational structure (e.g., Gentner et al., 2001), language is strictly impossible without analogy and the distinction between ‘literal’ and ‘analogical’ is one of degree. We favour an intermediate view, in which analogies exist along a spectrum, and commonly shared labels provide the raw material from which complex analogies involving the deliberate mapping of structural similarities between a novel concept and cultural reference can be made. We intentionally focus on the consequences of analogical communication – what analogies *do –* by modelling a specific functional mechanism: the use of structural similarity between familiar and novel concepts for the purpose of learning.

The emergence of language exacerbates a fundamental cooperative dilemma: language makes it easier to transmit complex, abstract information, but it also makes it easier to create complex and exploitative *lies* 2011). If communication is sufficiently untruthful, cooperative coordination is unlikely to develop (Crawford & Sobel, 1982). Costly indicators of aligned interests are a well-known means of ensuring the veracity of a signal, and therefore the long-run viability of communication (Higham, 2014; Smith & Bliege Bird, 2005). Analogies that rely on knowledge repositories shared only among members of cooperative groups with common interests may alleviate some of this conflict by providing a signal that is time-consuming to acquire and requires in-group teachers, making it difficult to counterfeit.

A proximate benefit of communicating novel information via analogy, drawing on structural similarities with shared knowledge, is a gain in efficiency. Compared with communication that minimally draws on shared knowledge and is instead maximally explicit and unambiguous to the widest range of possible listeners, analogical communication compresses a large amount of contextual detail and relational information into something clear and concise, at least for those listeners with the appropriate cultural background. Imagine a culture in which a specific hand-gesture is used to represent a bird called a ‘nockatoo’, especially during activities like hunting when quiet is important. This hand gesture consists of holding the index, second and third fingers straight whilst holding down the little finger with the thumb, representing the bird’s robust crest. Now imagine a completely separate skill – say, tying a complex knot – the mastery of which is aided by holding up three fingers and wrapping the string alternately around each finger. Teaching someone this knot is made easier if one can simply say something like ‘do a Nockatoo with your left hand’. The hand formation is, in this instance, completely unrelated to the bird, but provided the analogy is successfully interpreted, it will nevertheless cause the learner to hold their hand in the correct shape. The instruction compresses ‘hold your index, second and third finger up straight, whilst holding your little finger down with your thumb’ into a single word, and because the learner already knows how to make their hand into the Nockatoo shape, there is substantially less new material to reconstruct. Of course, this kind of analogical communication only works if two individuals have a shared repertoire of cultural information to draw from. If the listener in this example had never seen a Nockatoo or learned the associated gesture, the teacher would likely have to revert to slow, step-by-step description, using more explicit and less contextually tailored concepts that do not permit the same degree of information compression.

In this paper, we present formal models illustrating the feasibility of the idea that analogical communication facilitates high-fidelity social transmission, dependent on a sufficiently large shared cultural repertoire, and can evolve even when costly. For simplicity, we do not explicitly model the evolution of the shared repertoire, but we do consider cases involving repertoires of varying size. We show that (a) even a small shared repertoire brings benefits, and (b) the benefit of analogical communication increases with repertoire size. Thus, runaway selection on repertoire size is possible once the cognitive and cultural mechanisms facilitating shared repertoires came into being. Further, the emergence of analogies then scaffolds more and richer analogies.

We model a system in which agents must find behavioural solutions to a variety of problems. Like many social learning models, agents can inherit solutions from the previous generation, and can learn socially or asocially to solve problems (Aoki & Feldman, 2014; Boyd & Richerson, 1985; Kendal et al., 2018; McElreath et al., 2013; Rogers, 1988). However, our model differs from previous social learning models in at least three important ways. First, while most models blackbox the learning process and assume that individuals either do or do not acquire adaptive solutions, we explicitly model learning as a search in a rugged, multidimensional solution space in which various solution elements may be interdependent. This allows us to capture graded variation in the quality of social learning, as well as variation in the complexity of problems. We specifically model solution space as an NK landscape. Originally developed as a model of fitness resulting from epistatic gene regulatory networks (Kauffman & Weinberger, 1989), the NK landscape has more recently been used to model search for behavioural or technological solutions using both individual and social learning (Barkoczi & Galesic, 2016; Boroomand & Smaldino, 2021, 2023; Gomez & Lazer, 2019; Lazer & Friedman, 2007; Wu, 2024). The model has two key parameters: *N* is the number of components a solution can have (the number of dimensions in the solution space), while *K* is the typical number of interdependencies between solution components (the ruggedness of the fitness landscape). More details are provided in the next section. The NK landscape model also produces results consistent with several other models of problem-solving, indicating that it is robust (Smaldino et al., 2024).

Second, while most NK landscape models assume that individuals are searching for the best solution to a single problem (represented by a single NK landscape), we consider scenarios in which individuals must find solutions to *many* problems throughout their lifespan, by simulating *many* landscapes simultaneously. This allowed us to represent analogies as opportunities for teachers to guide learners to the solution to a novel problem (a new NK landscape) by invoking similarities to known problems and their known solutions. As far as we know, modelling agents who must use their knowledge to solve multiple NK landscapes has not been done previously, at least in the context of problem solving or social learning.

Third, many models of social learning assume that information is transmitted perfectly, or with some error, from one individual to another. This is a useful modelling assumption, but has been criticised by some cognitive scientists for failing to account for nuances in the representation and transmission of information (Claidière & Sperber, 2007; Heyes, 2016; Miton & DeDeo, 2022; Sperber, 1997). In our model, we address this concern and assume that complex information cannot be fully transmitted wholesale from one mind to another. Instead, teachers employing analogical communication to share their solution with a learner will transmit *the most similar solution in their shared repertoire* to the new problem. In other words, teachers will communicate that the solution is similar to something the learner already knows. By pointing out analogies to features in shared knowledge, teachers can dramatically improve the likelihood that the learner discovers a good solution to the new problem. All analogies are ultimately imperfect (French, 1995), and the student still has to complete or refine the given solution with their own asocial learning to reach the correct solution. This framing is similar to a recent model of teaching via tacit learning (Miton & DeDeo, 2022), which we view as complementary. The proposed process represents a combination of analogical teaching, aided by (proto)language, and individual learning with feedback from the environment or a teacher, as opposed to pure imitation/copying. We believe this represents a realistic scenario of human cultural learning following the emergence of protolanguage.

We structure our presentation into two models illustrating our proposed theory. The first model concerns individual pairs of learner and teacher, in which the learner must find the solution to a complex novel problem. The teacher can use analogy to guide the learner’s search towards a solution to a similar problem. In this first model, we take the ability to use analogy as given, and focus on its use and the size of the shared repertoire to improve problem solving on the part of the learner. The second model considers a population of learners and students, in which the ability to use analogies is costly and must evolve in a population that does not use analogical communication. This allows us to directly investigate the evolutionary plausibility of our argument.

It is worth noting that individuals must have already established sufficiently effective communicative abilities to maintain a shared repertoire of knowledge that can be drawn upon for use in analogical communication. A shared environment can potentially account for some amount of shared knowledge (Kline, 2015), but other knowledge, especially knowledge that involves shared categories, narratives, and abstractions, requires analogy (Brand et al., 2021). We surmise that as competency with analogical communication grew, so did the potential for ever larger shared repertoires. Ultimately, further modelling must explain how the maintenance of a shared repertoire *coevolved* with the ability for analogical communication. For now, we allow ourselves to assume the existence of the shared repertoire (aka cultural information) to explore how shared knowledge of this type facilitates the use of analogy to communicate novel complex information.

## Methods

We first describe a model involving a single teacher–student pair in which the student learns a solution to a novel problem using analogy, which we use to illustrate how analogical communication provides an advantage in the communication of complex information. We then expand the model to consider a population of agents solving multiple problems over many generations, with vertical transmission of solutions and learning style, in order to explore the conditions under which the cognitive machinery for analogical communication can evolve even when costly.

We use ‘analogy’ throughout to specifically refer to the deliberate mapping of relational structure from a familiar concept, shared between learner and teacher, to a novel target concept. We acknowledge that the boundary between analogy and other aspects of language is not always clear-cut, especially since constructs such as metaphor or allusion may be viewed as distinct from analogy by some scholars. We rely on the model formalism to indicate the type of relationships we are focusing on; it is a benefit of formal modelling to alleviate some of the ambiguity inherent in purely verbal explanations (Frankenhuis et al., 2023; Smaldino, 2017). We operationalise the analogy formation process by using the Hamming distance between two solution vectors to identify structural similarity between the target problem and shared knowledge (described in more detail below). We thus avoid making strong assumptions about the cognitive processes by which analogies are constructed in order to focus on the downstream consequences of analogical communication.

In both models, NK landscapes are used to model complex problems that require complex solutions (see Csaszar, 2018; Boroomand & Smaldino, 2021 for primers on NK landscapes). Solutions are represented as bit-strings of length *N*. Each of the *N* positions (bits) on the bit-string is associated with a score depending on its own value (0 or 1), and the value of *K* other ‘interdependent’ positions, so that *K* is a measure of interdependence or problem complexity (0 ≤ *K* < *N*). The total score of the solution is calculated by summing the score for each position and dividing by the length of the bit-string, *N*, so that each unique bit-string has a score associated with it. Each of the 2*^N^* possible solutions corresponds to a position on the landscape, with a score for each position stored in a lookup table.

An individual agent begins with a random solution and then searches for better solutions using a simple ‘hill-climbing’ strategy. One randomly selected bit of their solution is changed, and the agent determines whether this change has increased or decreased the score. The new solution is then accepted with a probability proportional to the difference in score between the new and old bit-string, and becomes the agent’s new solution from which further search will occur. We use NK landscapes with *N* = 20, meaning that each landscape represents approximately one million possible solutions. We then consider two types of problem complexity, with *K* = 0 representing simple, easy-to-solve problems (because each bit position affects the solution score independently), and *K* = 10 representing complex, difficult problems (these parameters have also been used in prior work to represent simple and complex problems; Barkoczi & Galesic, 2016; Boroomand & Smaldino, 2021, 2023). It is very difficult to find the optimal solution to complex problems with simple hill climbing due to the high interdependency of the bits. Larger *K* values produce rougher landscapes with many suboptimal solutions that have moderately high scores and are difficult to improve upon via hill climbing; smaller *K* produce smoother ‘landscapes’ which are easier to optimally solve in this way. Hill climbing is often thought to represent a largely individual process of trial-and-error learning. Past work has shown that communication among agents with diverse knowledge can improve the probability that a group or team of agents achieves a close-to-optimal score (Barkoczi & Galesic, 2016; Boroomand & Smaldino, 2021, 2023; Gomez & Lazer, 2019; Lazer & Friedman, 2007; Shore et al., 2015; Smaldino et al., 2024; Wu, 2024; Yahosseini & Moussaïd, 2020). Our model design is consistent with other recent work highlighting the value of social learning in guiding and constraining subsequent individual exploration (Ehn & Laland, 2012; Enquist et al., 2007; Turner et al., 2023).

## Individual pairs of agents

We first consider a pair of agents in which one agent (the teacher) has a skill that the other agent (the student) is tasked with learning. The problem to be solved is represented by an NK landscape with *K* = 0 (an easy problem) or *K* = 10 (a difficult problem), and the skill by a bit-string (solution) to that landscape. The student and teacher both have knowledge of a pool of shared solutions which have already been learnt, the *shared repertoire*. We assume that, within any group, there is a collection of basic knowledge, skills, stories, and other culturally specific information that is learnt by everyone in the group during development. We do not explicitly model the acquisition of the shared repertoire. Instead, teacher and student agents are initialised with a set of *R* randomly chosen solutions (bit-strings of length *N*), held in memory. The size of the shared repertoire (*R*) is varied between 0 and 10^5^, with a default value of 10^3^ (see Table 1 for a list of model parameters and their default values).

**Table 1.** Model parameters and their default values for the pair and population models Table 1 long description.

Symbol	Definition	Default value (pair model)	Default value (population model)
*N*	Number of bits in a solution	20	20
*K*	Interdependency of bits (landscape complexity)	10 (difficult-to-solve), 0 (easy to solve)	10 (difficult-to-solve), 0 (easy to solve)
*R*	Shared repertoire size	1000	1000
*T*	Hill-climbing iterations	200	200
ε	Hill-climbing exploration rate	0.001	0.001
*c*	Repertoire cost	N/A	0.0005
	Problem pool size	N/A	100
*e*	Rate of environmental change	N/A	0.05
*X*	Population size	N/A	100
*m*	Mutation rate (analogy to non-analogy)	N/A	0.01

An analogy is found by searching the shared repertoire for the solution which is most similar (determined by the smallest Hamming distance) to the teacher’s solution. The student then begins hill climbing from this solution. When *R* = 0, no analogy learning is possible because the teacher and student have insufficient overlapping knowledge, and instead the student must learn individually, starting from a random solution. Conceptually, this represents the baseline condition in which the mechanism we are studying (the mapping of structural similarity from the shared repertoire to a novel domain) is absent. Learners who lack a sufficient shared repertoire with their teacher must rely on individual trial-and-error learning, though their initial random starting position could hypothetically be acquired in any number of ways not modelled here. The model is agnostic with regard to which agent selects the analogy. For example, the teacher might use their knowledge of the solution to choose a suitable analogy, the student might observe the teacher and recognise structural similarities between the new problem and a skill in the shared repertoire, or a combination of the two may occur – these are all equally valid interpretations. We ‘black-box’ the process of analogy formation, including the cognitive processes that allow the identification and mapping of structural similarities between two concepts. It is taken as a given that a) humans possess this ability when *R* > 0, and b) that in identifying a suitable analogy some information about the solution is gained. This allows us to instead focus on the downstream implications for learning and skill transmission.

We ran simulations using easy- and difficult-to-solve problems, with different repertoire sizes (with *R* = 0 corresponding to the absence of analogy), in which students had 200 hill-climbing iterations to find their solutions. Visual inspection of our simulation results indicated that this was typically plenty of time for learners to settle on a solution from which they did not further deviate. For each simulation run, a different NK landscape was chosen at random to represent the novel problem, from a set of 1000 randomly generated NK landscapes with pre-computed optimal solutions. The purpose of this model is to illustrate the advantage of analogical communication in the ability to discover solutions to complex problems.

## The population model

In order to study the evolution of analogical communication, we extended the pair model to consider a population of agents over many discrete generations. The population model differs from many typical NK landscape social learning models in that we generate multiple NK landscapes and allow our agents to share solutions within and between these different landscapes. Agents can be analogical learners, or ‘non-analogy’ learners. We use ‘non-analogy learners’ as a convenience to refer to agents who do not draw on structural similarities between novel problems and concepts in the shared repertoire. We leave it to the reader to consider the broader philosophical question of whether truly non-analogical language exists. When an agent encounters a novel problem, those who learn via analogy obtain the most similar solution from the shared repertoire and continue hill-climbing from there. The shared repertoire, as in the previous model, is created at the start of a simulation by randomly generating *R* bit-strings, and is shared by all agents in the population.

Each agent has a single (either cultural or genetic) parent who acts as their teacher, and each parent can have zero or multiple offspring (students). The number of offspring an agent has is determined with a probability proportional to the agent’s reproductive fitness. Fitness is, in turn, determined by the solutions each agent finds to the problems in their *problem set* of 10 NK landscapes. Each simulation uses a pool of 100 NK landscapes, from which 10 are selected as the ‘problem set’ for each generation. These represent problems/opportunities in the current environment, and the solutions to these problems represent skills or technologies that confer some fitness advantage. In each generation, there is a fixed per-problem probability of environmental change, *e*, that renders a skill maladaptive or obsolete. When this happens, the problem is replaced by a new one drawn from among those problems in the pool for which there is no prior knowledge in the population. For the default of *e* = 0.05, there is a probability of 0.4 that at least one of the 10 problem set landscapes will be novel in any given generation, corresponding to the high rates of environmental and/or social change in which most recent human evolution occurred (Foley & Gamble, 2009; Jones, 2011; Richerson & Boyd, 2000). For computational tractability, we pre-computed optimal solutions to 1000 NK landscapes, and each simulation generates its problem pool from these.

How each agent obtains solutions to the problems in their problem set depends on two things: whether the problem existed in their parent’s generation, and whether they are an analogy user. If the problem existed in their parent’s problem set, agents faithfully copy their parent’s solutions. We imagine that this high-fidelity transmission can happen gradually during development, so that there is ample time for feedback and correction. If the problem is new (due to environmental change) and therefore unknown to the agent’s parents, the agent learns via hill climbing. An agent has a fixed amount of time, *T* time steps, for hill climbing, which is divided evenly among all novel problems. E.g., if there are two novel problems, they will have *T*/2 time steps to find a solution for each problem. The specifics of this hill-climbing period depend on whether or not the agent is an analogy user. For non-analogy users, hill climbing starts by selecting a random solution from their repertoire and continues for *T*/*n* time steps, where *n* is the number of novel problems facing the agent (we also tested a version where hill climbing started from a completely random location, with no qualitative change in results). The agent’s solution to a problem is the best solution encountered during hill climbing. If the agent is an analogy user, then they can draw an analogy between the novel problem they are faced with and the set of known problems in their shared repertoire, which includes any solutions discovered by their parent. An analogous solution is selected from the shared repertoire in the same manner as in the single-pair model, by finding the stored solution most similar (with the smallest Hamming distance) to the optimal solution for the novel problem. Because the parent transmits acquired knowledge that does not include a solution to novel problems, our framing that the student starts their search from the closest solution in the parent’s repertoire can be interpreted in at least two different ways. First, the parent could acquire novel information later in life, and use analogy to prior knowledge to communicate that with the learner. Second, the parent could remain ignorant of the new problem, and the learner could themselves discover an analogy to previously learned scenarios. Both of these scenarios are likely to occur, and we do not differentiate between them here. Once an analogous solution has been found, the agent starts hill climbing from there.

Because, all things being equal, learning via analogy provides an advantage to the learner, we explored whether the size of that advantage could outweigh costs that increase with the size of the shared repertoire. Such costs could be incurred in terms of the increased metabolic demands of a larger brain needed for such a memory or the developmental time needed to acquire detailed cultural knowledge (Stout & Hecht, 2017; Boyd & Richerson, 1996; van Schaik & Burkart, 2011). Either way, analogy-using agents may have fewer hill-climbing iterations available to them than a non-analogy user. The total time available for hill climbing becomes T* = *T* − *cR*. We also explored the effect of direct fitness costs, in which a cost was subtracted from the analogy user’s fitness. The results are qualitatively the same (see Figure S2). Even if analogical communication does not involve any additional costs, as in the case where analogical reasoning utilises cognitive abilities that evolved in response to unrelated selection pressure, considering them serves as a stronger test of our argument’s viability.

The population model is initialised with *X* = 100 agents, 5% of which learn with analogy in the first generation and the rest of which use regular non-analogy learning. Importantly, all agents use *some* amount of social learning to inherit their parents’ repertoire. At initialisation, there are no parents to learn from, so each agent must individually learn a new solution using hill climbing for each of the 10 ‘problem set’ landscapes. Agents inherit their parent’s learning style (analogy or non-analogy), though there is a small probability, *m*, that an analogy user will ‘forget’ their analogy-making ability and revert (or mutate) to regular non-analogy learning. Non-analogy users never mutate into analogy users. This ‘forgetting’ risk represents the extra evolutionary/cognitive cost for analogy users, perhaps reflecting the cognitive or memory load needed for making analogies. As a result, if analogy does not provide a fitness advantage, it will eventually disappear from the population via drift.

We simulated each population for 200 generations, which was more than sufficient time to reach fairly stable rates of analogical learning. We tracked the proportion of analogy users in the population, as well as their fitness, based on changes to shared repertoire size, *R*, and to the environmental turnover rate, *e*. Python code for our simulation model and C++ code for generating and solving NK landscapes is available at https://github.com/lottybrand/analogy/releases/tag/Accepted.

## Results

### Pairs of learners

**Figure 1.**
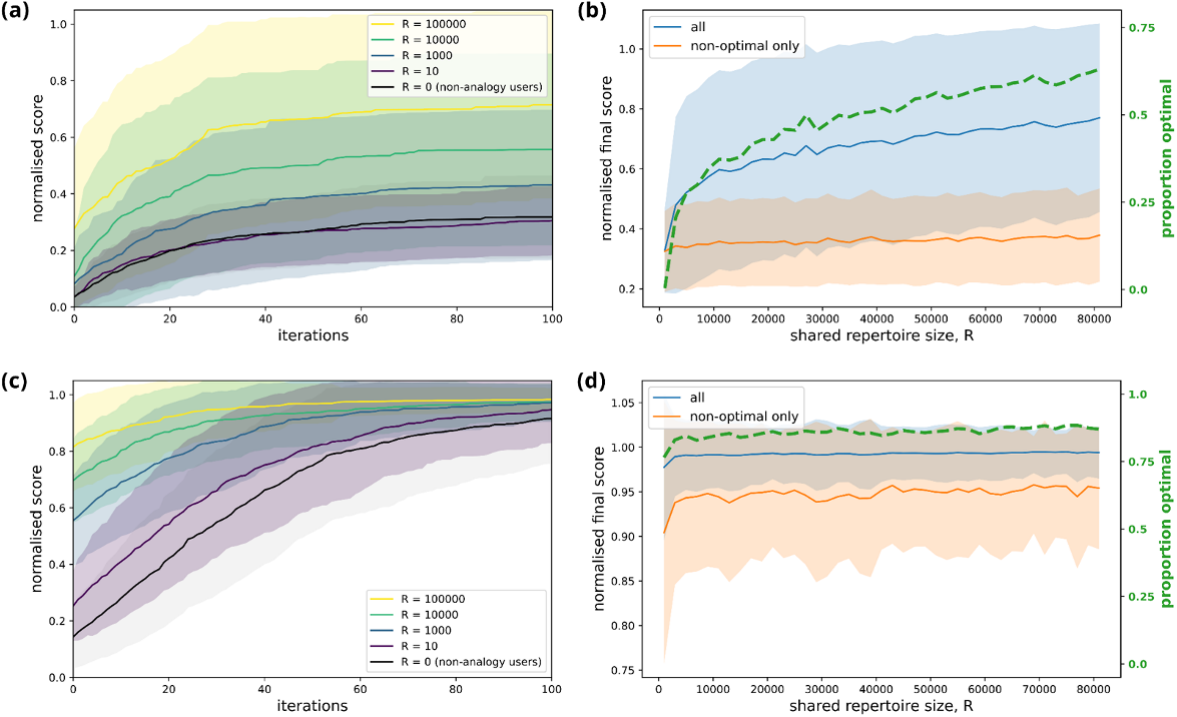
(a) Time series of mean student scores across hill-climbing iterations, for different shared repertoire sizes (*R*). Larger *R* led to higher average solution scores for difficult-to-learn problems (*K* = 10). All scores were normalised by the globally optimal score for the landscape being learnt. (b) The normalised final student scores (left axis) and proportion of globally optimal solutions found (green dashed line, right axis) for different shared repertoire sizes. The mean final score increases with repertoire size because a greater proportion of agents find the optimal solution. There was no improvement in the scores of agents which did not find the optimal solution. (c) Time series of mean student scores across hill-climbing iterations for smooth easy-to-learn landscapes (*K* = 0). Agents were able to find optimal or near-optimal solutions regardless of shared repertoire size, although larger shared repertoires were associated with faster convergence. (d) For *K* = 0, larger repertoire sizes were not associated with higher proportions of agents finding the optimal solution nor higher average solution score. Note that the non-analogy scenario is equivalent to when *R* = 0. Mean scores were calculated from 2000 repeat simulations, and shaded regions show ±1 standard deviation. Figure 1 long description.

We first considered the situation in which learning takes place as a one-off event between a teacher and a student, in isolation from other environmental or evolutionary dynamics. We assumed the teacher knows the optimal solution to a novel problem, and compared the student’s solution quality for different shared repertoire sizes and easy- and difficult-to-solve problems. The effect of shared repertoire size (*R*) varies depending on the complexity of the problem being solved. For complex problems (*K* = 10), larger *R* led to higher average solution scores. The time series of mean student solution scores is shown in Figure 1a for different repertoire sizes. Interestingly, the positive relationship between mean final score and repertoire size is driven by an increase in the proportion of agents finding the optimal score, rather than a more general improvement in solution score. That is, the scores of agents not reaching the optimal solution did not seem to improve with larger repertoire size (orange line in Figure 1b). This suggests that, at least for these complex environments, there may be a threshold of analogy quality, where a good analogy can help a student find a path to the optimal solution, but less good analogies are not much better than starting at random and can easily lead you down the wrong path.

For easy-to-solve problems (*K* = 0), the majority of students were able to find the optimal solution regardless of shared repertoire size (Figure 1d), although larger shared repertories led to faster convergence (Figure 1c). For both easy- and difficult-to-solve problems, a larger library of shared solutions meant that a learner was more likely to find an analogous solution similar to the novel problem’s solution. For easy problems, where the student could likely reach the optimal solution eventually through individual learning alone, a good analogy reduced the number of hill-climbing iterations needed to get there. For difficult problems, where there are lots of local maxima in solution space, a good analogy made it more likely that subsequent hill-climbing reached the optimal solution, as opposed to one of these local maxima. The relationship between repertoire size and mean final score shows diminishing returns in both cases, with increases in repertoire size for large repertoires conferring less additional benefit than the same increase at lower repertoire sizes.

**Figure 2.**
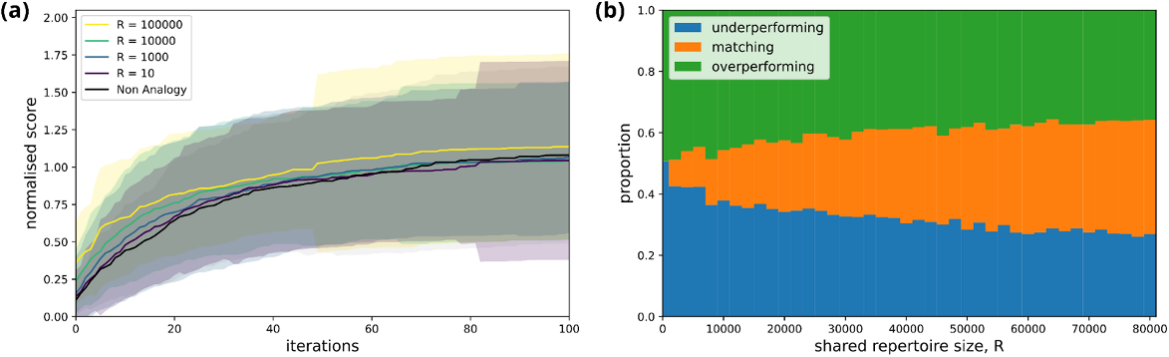
Teachers hill-climbed their solutions to difficult-to-learn problems (*K* = 10) before attempting to teach them to the student. (a) Larger shared repertoires lead to faster convergence on the teacher’s solution, but not higher average scores. Scores have been normalised by the teacher’s score. Mean normalised scores were calculated from 2000 repeated simulations, and shaded regions show ±1 standard deviation. (b) Larger shared repertoires increased the proportion of students that exactly matched the teacher’s solution (orange region), with a corresponding decrease in the proportion of students who over- and under-performed the teacher’s solution (blue and green regions). Proportions were calculated from 2000 repeat simulations for each shared repertoire size used. Figure 2 long description.

To clarify whether analogies are simply helping agents to quickly and accurately learn their teacher’s solution to complex problems (*K* = 10) or whether analogies actually allow them to further innovate on the teacher’s solution, we repeated the above simulations so that teachers initially hill-climbed their own solutions instead of using the global optimal. We did not perform these simulations for the easy-to-solve landscapes, since agents were able to find optimal solutions for these problems easily, even without the shared repertoire (i.e., *R* = 0). Analogies were therefore selected by comparing them to the teacher’s hill-climbed solution, instead of the global optimal. This made it possible for students to exceed their teacher’s score. Shared repertoire size had no impact on the final score (Figure 2a). Larger shared repertoires did improve students’ ability to learn their teacher’s solution more quickly and at a higher rate, while decreasing the proportion of students who found solutions that over- or under-performed their teacher’s score (Figure 2b). Shared repertoire size therefore acted similarly to an exploration–exploitation tradeoff parameter, with large repertoires leading to reliable convergence to the teacher’s solution, and small repertoires leading to greater variation in the quality of solutions found. This ‘tightening’ around the teacher’s solution could potentially limit innovation for large repertoires, though it is likely that other mechanisms not included in our model (like occasionally ignoring social information, or communicating with individuals from outside one’s community) were more important drivers of innovation (Fogarty et al., 2015; Smaldino et al., 2024).

The proportion of students who matched their teacher’s solution was also sensitive to the hill-climbing exploration rate. Larger exploration rates, which allow agents to better escape local optima, made convergence to the teacher’s solution less frequent, with a corresponding increase in both over- and under-performing students (Figure S1).

Overall, these results suggest that learning with analogies may serve to enhance the accuracy of social transmission, reduce the number of individuals with very bad solutions, and reinforce homogeneity by limiting exploration. Analogical communication does not necessarily improve a student’s ability to reach an optimal solution if the teacher does not already know this solution, a result which suggests an important role for domain experts to serve as teachers or demonstrators (Henrich, 2004). This is not to say that analogical reasoning does not play a role in creative problem solving – it almost certainly does (Holyoak & Thagard, 1995). We are instead highlighting the adaptive feature of analogy in improving communication fidelity.

#### Population dynamics

Next, we considered population dynamics of agents and the evolution of analogical communication. In this section, we return to our original method in which analogies were selected by comparison of shared repertoires to the optimal solution. This can be interpreted as the analogy-using learner having access to an expert in the target skill/behaviour, either as an observer or through direct teaching. Due to the benefit conferred by analogy in terms of social transmission, analogy use evolved as long as that benefit was not outweighed by cognitive, behavioural, or metabolic costs required to facilitate analogy.

**Figure 3.**
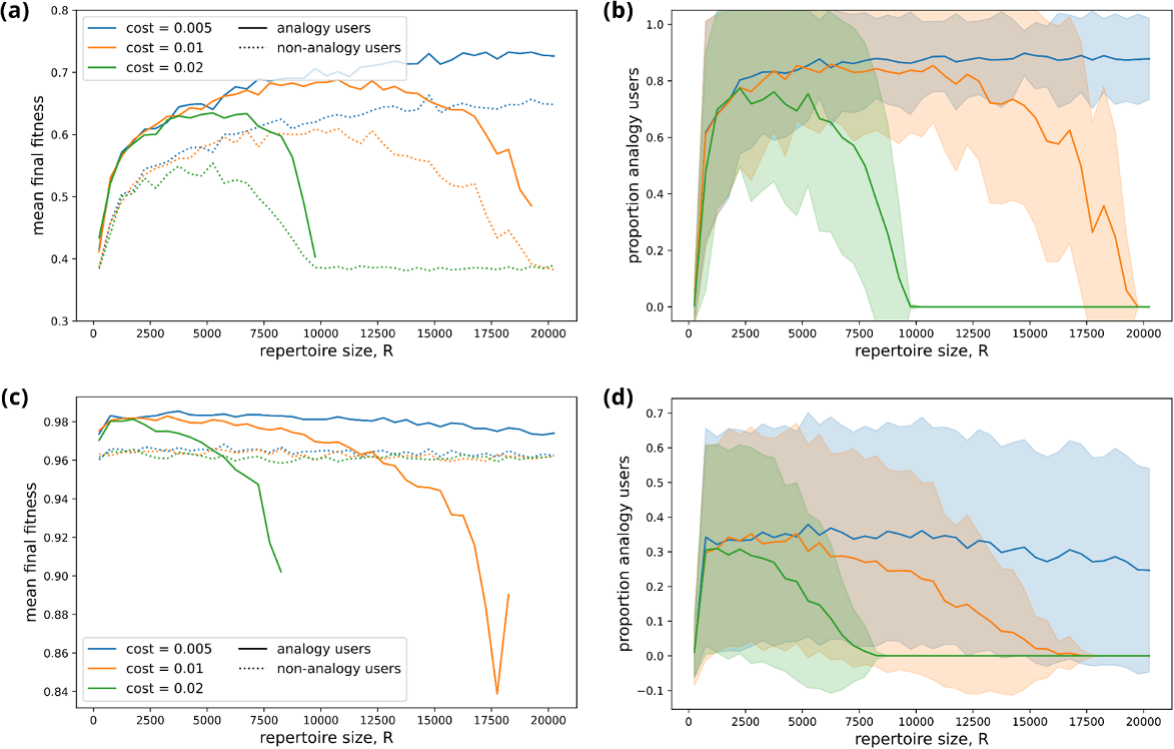
Simulations for a population of agents, where learning style (analogy or non-analogy) was inherited, and there was a small cost associated with analogy use. In (a) and (b), difficult-to-solve problem sets were used (*K* = 10). (a) shows the time series of mean final fitness and (b) shows the mean proportion of analogy users. Moderately sized shared repertoires were associated with higher mean population fitness. Mean fitness for analogy users and non-users has been plotted separately, and the difference between these two groups was also greatest at intermediate shared repertoire sizes. The mean proportion of analogy users reflected the pattern in mean fitness, with intermediate shared repertoire sizes resulting in the highest proportion of analogy users. Shaded regions show ±1 standard deviation. Means and standard deviations were calculated from 250 repeat simulations over 200 generations for each combination of parameter values. (c) and (d) again show the mean fitness and proportion of analogy users for different shared repertoire sizes and costs, but this time for easy-to-solve problem sets (*K* = 0). Smaller shared repertoire sizes were always associated with higher mean fitness. Figure 3 long description.

For difficult-to-learn problem sets (*K* = 10), larger shared repertoire size was associated with greater mean population fitness (Figure 3a) and therefore resulted in larger proportions of agents using analogies (Figure 3b), up to a point. However, the diminishing returns in the benefit that larger repertoires confer, combined with the linearly increasing cost of acquiring the shared repertoire, led to a point where the cost associated with maintaining a very large repertoire outweighed the benefits associated with having it. This resulted in an ‘n’-shaped relationship between repertoire size and fitness, with small repertoires conveying only a limited advantage and large repertoires being too costly. The difference in mean fitness between analogy and non-analogy users was highest for mid-range repertoire sizes, although analogy use spread even at low repertoire sizes due to the fitness advantage of analogy users over non-users (by *R* = 500, the mean proportion of analogy users was already 0.5 for all three costs). The proportion of analogy users in individual runs was typically either zero or close to one, as analogy use either went extinct or spread to near-fixation depending on the cost of the shared repertoire. The average proportion of analogy users depicted in Figure 3b is therefore approximately the proportion of runs in which analogy use went to fixation rather than going extinct. We also repeated these simulations using a direct fitness cost, rather than a penalty to the number of hill-climbing iterations available. The results were equivalent, shown in Supplementary Figure S3.

Interestingly, while the mean fitness of non-analogy users was always lower than that of analogy users, they exhibited the same ‘n’-shaped relationship with repertoire size as the analogy users. This was due to diffusion of solutions acquired by analogy into the non-analogy-using population due to analogy users switching to non-analogy. This could be beneficial in the short term, since it allows agents to avoid paying the costs of analogy use, but as time passed and the problem set changed, they fell behind analogy users. This effect is demonstrated in Supplementary Figure S2a and b, in which the mutation rate was turned off, preventing solutions originally learnt by analogy users from entering the non-analogy population, which led to no change in the fitness of non-analogy users with changing shared repertoire size.

Next we considered the same cost scenario using easy-to-learn problem sets (*K* = 0). While analogy users with small shared repertoires had higher mean fitness than non-users, we found that the mean fitness of analogy users always declined as shared repertoire size increased (Figure 3c). This suggests that a population that needs to solve only simple problems would experience no positive selection for larger shared repertoires. Analogy use did spread in these populations, but remained in the minority, with the mean proportion of analogy users never exceeding 0.35 even for the lowest cost scenario, and tending to decrease as the shared repertoire size increased.

**Figure 4.**
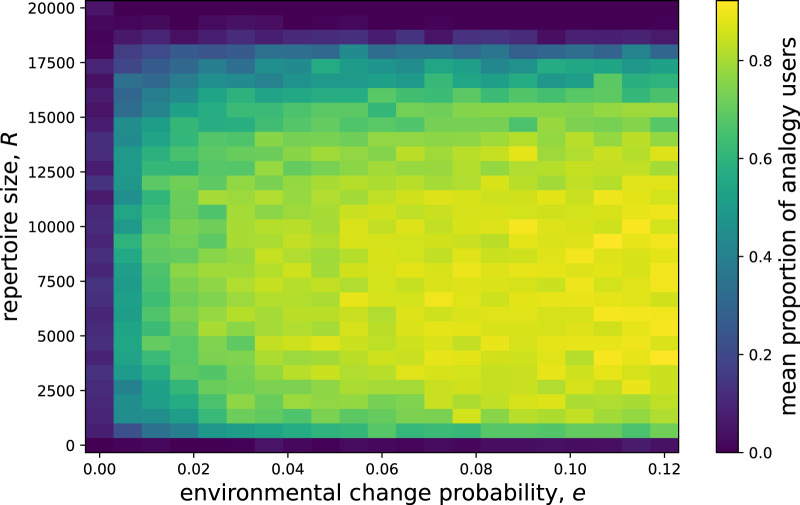
The effect of environmental change (which generates novel problem set problems) on the proportion of analogy users. The proportion of analogy users increased as the environment became less stable. More temporally variable environments can therefore support larger shared repertoire sizes. All other parameters were set to their defaults (Table 1). Each cell is the average of 100 repeat simulations, each with a population of 100 agents for 200 generations. Problem sets were comprised of 10 difficult-to-solve NK landscapes (*N* = 20, *K* = 10). Figure 4 long description.

Finally, we examined the effect of environmental unpredictability as a source of novel problems on the spread of analogy use. To do this, the rate of environmental change, *e*, was varied between 0 (constant through time) and 012. We considered only difficult-to-solve problem sets (*K* = 10) for this analysis because the easier problem sets did not require analogy learning to find optimal solutions. Unstable environments were generally associated with lower mean fitness because there was less time for especially good solutions to spread throughout the population (Figure S4). As expected, when the environment was very stable, the proportion of analogy users in the populations tended to zero. This is because, as modelled here, the primary benefit of analogies is to increase the ability of teachers to transmit detailed information for novel complex problems. In very stable environments, agents can get by with copying their parent’s solutions (which does not necessarily require analogical communication over developmental time) and thereby avoid paying the cost of acquiring the shared repertoire. This is consistent with prior work showing that costly social learning strategies tend to be favoured in more variable environments (Aoki et al., 2005; Rogers, 1988). Higher rates of environmental change were associated with more widespread analogy use in our analyses, because there were more opportunities to benefit from using analogies for a given cost of acquiring the shared repertoire (Figure 4).

## Discussion

The proximate use of analogies in thinking and communicating is well established. Indeed, analogy may be, as Hofstadter and Sander (2013) suggest, the ‘fuel and fire of cognition’. Here, we explore more ultimate functions for analogy in communication in our evolutionary (or at least cultural evolutionary) past, building on verbal arguments laid out by Brand et al. (2021), and demonstrating how analogical communication could have played key roles in maintaining the fidelity of social transmission for complex information. In this way, we also make progress towards opening the ‘black box’ of social learning (Heyes, 2016) by considering what sorts of cognitive mechanisms actually facilitate high-fidelity cultural transmission.

Our focus on the functional aspects of analogy is both a strength and limitation. Our formal use of Hamming distance to operationalise the search for good analogies retains a level of abstraction that makes our results broadly applicable to phenomena involving the mapping of similarity from one domain to another for the purpose of learning, without being tied to a specific definition or cognitive model of analogy. Our results are therefore relevant whether adopting a permissive ‘all categories are analogies’ definition (e.g., Hofstadter & Sander, 2013) or a narrower one requiring deeper mapping of relational structure between concept domains (e.g., Gentner et al., 2001). Our non-analogy (*R* = 0) scenario can be understood as a lower bound on analogical reasoning rather than its complete absence. The cost of this generality is that we can only comment on the downstream consequences of analogy; the model cannot distinguish between competing definitions of analogy. We focus our discussion on those aspects oriented towards the communicative transfer of complex relational structure.

Our analysis suggests that analogical communication enhances the fidelity of social transmission, and can evolve even if costly, provided that there exist sufficiently complex problems that cannot easily be solved through individual learning alone. Once emerged, the ability to communicate using analogy, even with a small initial shared repertoire, could scaffold the development of ever-larger repertoires among members of social groups. Our model suggests that increasing the size of a group’s shared repertoire further increases the fidelity of social transmission, though with diminishing marginal returns as the size of the repertoire grows. The ultimate size of the repertoire may be constrained by cognitive limitations such as memory capacity (Ghirlanda et al., 2017), developmental limitations such as the time needed to learn cultural information (Heyes, 2018), or pragmatic limitations such as the need to effectively coordinate with others on a limited number of options (Skyrms, 2010).

High fidelity social transmission is likely necessary for cumulative cultural evolution (Henrich, 2010; Boyd, 2018; Tomasello, 1999). Without forces that help steer utterances into consistent shared meanings (e.g., language), cumulative cultural evolution will be limited. Analogies are one likely mechanism for maintaining the fidelity of transmission of complex skills. Because analogies specify shared properties and relationships between two systems, they constrain and shape what is likely to be assumed and explored about the novel system. Along these lines, Miton and DeDeo (2022) recently presented a model that showed how preserving a small number of relationships in a complex interconnected system could stabilise cultural transmission better than pure imitation. We interpret their results as supporting our conclusions here with a substantially different model design. That said, *too* much consistency reduces the variation that is necessary for any type of evolution, cultural, or otherwise (Fisher, 1930). A lack of diversity can similarly hinder creative collective problem solving (Smaldino et al., 2024). However, we view it as unlikely that analogy usage in communication creates this problem. Miton and DeDeo (2022) showed that the communication of relational constraints (which we can interpret in terms of analogical communication) could occasionally generate creative leaps in idea space. More generally, outside of the sort of rigid formalism found in mathematics, symbolic communication almost always contains some amount of ambiguity (Eisenberg, 1984; Fitch, 2010), which can provide fuel for differential interpretation and thus creative exploration.

We did not explicitly model the development and emergence of shared cultural repertoires. However, the emergence of shared concepts need not be a particular puzzle. Once cooperative, communicative individuals with the basic abilities for categorisation and imitation live together in groups, the cognitive machinery that categorises stimuli and uses those categories to produce utterances is sufficient to produce the emergence of collectively shared categories (Falandays & Smaldino, 2022). This idea is consistent with more general theories of language evolution, which propose that protolanguage emerged and then culturally evolved once humans developed prosocial tendencies and imitative capacities (Chater & Christiansen, 2010; Kirby, 2017; Thompson et al., 2016; Tomasello et al., 2012). That said, some of this research has also suggested that certain aspects of shared knowledge, particularly those aspects that require repetition to learn, may be limited in their ability to spread in large ancestral populations due to the difficulty of diffusion across sparse networks (Reali et al., 2018), in a manner reminiscent of the sociological concept of ‘complex contagion’ (Guilbeault et al., 2018). Learning complex linguistic features like grammar is nontrivial. It is possible that even simple analogies could have aided the further cultural evolution of language by increasing the fidelity of social transmission. Indeed, what is language if not the ability to place analogical labels onto things, events, and scenarios, which relate them to other similar occurrences? In this spirit, we offer our model as an addition to the shared cultural repertoire of those of us interested in the evolution of language, culture, and communication, in hopes that, by analogy, it helps us to better understand those phenomena and others.

## Supporting information

10.1017/ehs.2026.10063.sm001 Holding et al. supplementary material Holding et al. supplementary material

## Supplementary material

The supplementary material for this article can be found at https://doi.org/10.1017/ehs.2026.10063.

## Research transparency and reproducibility interest

All model code, NK landscape implementation, and stored NK landscapes are available from https://github.com/lottybrand/analogy/releases/tag/Accepted.

## Table 1 Long description

Parameter defaults are listed for two related models: a pair model and a population model. Both models use the same settings for solution length, bit interdependency, shared repertoire size, hill-climbing iterations, and hill-climbing exploration rate. Solution length is 20 bits, shared repertoire size is 1000, hill-climbing runs for 200 iterations, and exploration rate is 0.001. Bit interdependency is given as either 10 for a difficult-to-solve landscape or 0 for an easy-to-solve landscape in both models. Only the population model includes additional parameters: repertoire cost set to 0.0005, problem pool size 100, environmental change rate 0.05, population size 100, and mutation rate 0.01. Entries marked not applicable indicate parameters that are not used in the pair model.

Navigate back to Table 1.

## Figure 1 Long description

Line graph A. The horizontal axis label is iterations, with a range from 0 to 100. The vertical axis label is normalised mean score, with a range from 0 to 1. The legend lists R equals 0, R equals 10, R equals 1000, R equals 10000 and R equals 100000. Each time series is a line with a surrounding shaded band. All lines increase from iteration 0 and then flatten. By iteration 100, the R equals 100000 line is the highest and the R equals 0 line is the lowest, with the other R values between them. Line graph B. The horizontal axis label is shared repertoire size, R, with a range from 0 to 80000. The left vertical axis label is normalised final score, with a range from 0 to 1. The right vertical axis label is proportion of globally optimal solutions found, with a range from 0 to 0.75. The legend lists all and non-optimal only. Two lines with shaded bands correspond to the left axis and one dashed line corresponds to the right axis. Across increasing shared repertoire size, R, the all line rises and then levels off. The non-optimal only line stays lower and is approximately horizontaly flat across the range. The dashed line for proportion of globally optimal solutions found increases across the range and approaches the upper part of the 0 to 0.75 scale. Line graph C. The horizontal axis label is iterations, with a range from 0 to 100. The vertical axis label is normalised mean score, with a range from 0 to 1. The legend lists R equals 0, R equals 10, R equals 1000, R equals 10000 and R equals 100000. Each time series is a line with a surrounding shaded band. All lines rise quickly and approach values near 1 as iterations increase. The separation between R values is smaller than in line graph A and the lines are closer together by iteration 100. Line graph D. The horizontal axis label is shared repertoire size, R, with a range from 0 to 80000. The left vertical axis label is normalised final score, with a range from 0 to 1. The right vertical axis label is proportion of globally optimal solutions found, with a range from 0 to 1. The legend lists all and non-optimal only. Two lines with shaded bands correspond to the left axis and one dashed line corresponds to the right axis. Across increasing shared repertoire size, R, the all and non-optimal only lines remain approximately constant with small fluctuations, although the all line is higher than the non optimal only line. The dashed line for proportion of globally optimal solutions found also stays nearly constant at approximately 0.87 with small fluctuations. Line graphs A and B use iterations and shared repertoire size, R, to show normalised score measures and the proportion of globally optimal solutions found for the same set of R values. Line graphs C and D use the same axis labels as A and B, respectively, with the same three plotted measures in the shared repertoire size, R, plots.

Navigate back to Figure 1.

## Figure 2 Long description

The first graph (a) plots normalized scores on the vertical axis against iterations on the horizontal axis. Normalized scores range from 0 to approcimately 1.5 and iteration range from 0 to 100. It includes multiple lines representing different shared repertoire sizes: 0, 10, 1000, 10000 and 100000.. The lines show an upward trend, with shaded regions indicating variability. The second graph (b) is a stacked bar graph showing proportions on the vertical axis and shared repertoire size R on the horizontal axis, ranging from 0 to 80000. It includes three categories: underperforming, matching and overperforming, with varying proportions across different repertoire sizes.

Navigate back to Figure 2.

## Figure 3 Long description

Four line graphs labeled (a), (b), (c) and (d). (a) and (c) depict the relationship between repertoire size, R, mean final fitness. (b) and (d) depict the relationship between repertoire size and proportion of analogy users. The x-axis for all four graphys is labelled ‘repertoire size, R’ and tanges from 0 to 20000. In graph (a), thethe y-axis is labeled ′mean final fitness′ and ranges from 0.3 to 0.8. The curves shows six curves, representing the mean final fitness for three costs: 0.005, 0.01 and 0.02, each for analogy users and non-analogy users. Analogy users generally having higher fitness at intermediate repertoire sizes. Graph (b) has the same x-axis but the y-axis is labeled ′proportion analogy users′ ranging from 0 to 1. There are three curves representing each of the costs. The curves indicate the proportion of analogy users, with shaded regions representing plus or minus 1 standard deviation. The proportion peaks at intermediate repertoire sizes. Graph (c) repeats the mean final fitness analysis for easy-to-solve problem sets . The curves start near 1 and slowly decrease, showing similar decreases at higher repertoire size with varying costs. Graph (d) mirrors graph (b) for different conditions, showing the proportion of analogy users, where the lines rapidly increase to approcimately 0.3 and then show downward trends as shared repertoire size increases. Shaded areas in all graphs represent variability across simulations.

Navigate back to Figure 3.

## Figure 4 Long description

Mean proportion of analogy users. Heatmap with a horizontal axis labeled environmental change probability, e, with tick labels 0.00, 0.02, 0.04, 0.06, 0.08, 0.10, 0.12. Vertical axis labeled repertoire size, R, with tick labels 0, 2500, 5000, 7500, 10000, 12500, 15000, 17500, 20000. A vertical scale bar at the right labeled mean proportion of analogy users, and ranges from zero to nearly 1. Across most repertoire size values from 0 to 20000, the mean proportion values are lower near environmental change probability 0.00 and become higher as environmental change probability increases toward 0.12. A band of lower values, indicating the proportion of analogy users is near zero, is visible when the repertoire size is zero, and along the highest repertoire size range near 20000 across the full environmental change probability range.

Navigate back to Figure 4.
